# Cholecystitis and Some of Its Sequelae

**Published:** 1913-07

**Authors:** A. F. Sampson

**Affiliations:** San Francisco, California


TEXAS MEDICAL JOURNAL.

Established July, 1885

F. E. DANIEL, M. D., - - - - - Editor, Publisher and Proprietor

Published Monthly.—Subscription, $1.00 a Year.

VOL. XXIX. AUSTIN, JULY, 1913. No. 1.

The publisher is not responsible for the views of the contributors.

For Texas Medical Journal.

### Original Articles.

### Cholecystitis and Some of Its Sequelae.

BY A. F. SAMPSON, M. D., SAN FRANCISCO, CALIFORNIA.

The consideration of appendicitis with its far-reaching ramifications in distressing the normal functions of the prima-via, etc., has been so deeply investigated that we find the trend of the medical mind now delving, and very becomingly so, into gall bladder troubles and its sequelæ for the relief of outraged nature. This subject carries extra potency to one who has been afflicted with gall stones, liver abscesses and concomitant disturbances resulting therefrom. My experience for many years in the observations of the functions of the gall bladder and liver have held my attention very closely. We will not endeavor to go into the detail actions of the many and various physiological functions of the liver, but give forth the results of some observations that must prove interesting.

The liver appeals to my sincerest appreciation, as this organ is called upon not only to play an essential part in its work to dispose of the chyle that is brought to it from the intestines, but its efforts in disposing of waste products of the animal economy is but a slight tax when we compare these, with its efforts to eliminate from the body the results of systemic infections and toxines. To those who live in the Southern States, where infections are more rife than in other sections of this country, the greeting, "How is your liver?" is as apropos as that of the Englishman in the East Indies.

In a very malarial section of South Carolina during the fall of 1883, I contracted a severe case of pernicious malarial fever. Shortly after this malarial infection, I suffered several attacks of gall stone colics. By the free use of quinine, etc., my recovery from gall bladder infections was accomplished, my general health and nutrition was fully restored.

That malarial infection is an etiological factor in cholecystitis can be no doubt. Enlarged livers and evidences of disturbed nutrition so prominent in those suffering from malaria can but claim attention to this cause in cholecystitis. In 1897 I recovered from an attack of yellow fever. The sequela from this infection was quite manifest by a cholecystitis terminating in such an accumulation of gall stones that a cholecystotomy was necessitated in 1906. It is interesting to observe the difference in the macroscopical appearance of gall stones from the various infections.

In considering the methods of treating gall stones and their sequelæ, my observations convinced me that proper ante and post operative attention should be given such cases. Frequently we find recurrence of the gall stones and a failure to attain a restoration of the patient's nutrition after the removal of the gall stones and drainage alone of the gall bladder. When we find gall stones we must not fail to appreciate the concomitant pathological conditions existing therewith, especially such as the infected and thickened mucosa of the gall bladder that often extends into the bile ducts, thereby impairing their patulency. My method is after the contents of the gall bladder has been well emptied, this is wiped out with a gauze and the ducts are probed out. When the drainage tube has been fixed and brought up as high as possible, I syringe through this a diffusible antiseptic solution such as Lugol's solution in saturated solution of boracic acid, repeating this twice a day until convinced that by this treatment we have overcome the infection of the lining of the gall bladder and the thickening of the ducts.

It has been very satisfactory to note since making such post operative treatment, I have not had a recurrence of gall stones nor trouble with the ducts, patients being restored to their normal functions. To demonstrate this point of the necessity of the post operative treatment, will cite but one interesting case where a cholecystectomy was done.

Mr. F., of Portland, Oregon, whom I met in consultation with Dr. Simon Baruch at Paso Robles in the spring of 1906. We found his condition called for immediate gall bladder surgical procedure. Next morning the patient returned to his home in Portland, Oregon, where he underwent a cholecystectomy. Within a year after he applied to me here for relief of pathological conditions fully as distressing as when I had previously seen him.

Upon reopening the gall bladder it contained vitiated bile, all its coats thickened. No gall stones were found nor were any stones found in the common bile duct, but a very thickened mucosa, also probing in the bile ducts revealed almost an impatulous state of the common bile duct.

Along the lines of post operative treatment as above stated, this patient made a perfect recovery, and though five years have elapsed, he has experienced no pathological disturbance. I have seen him from time to time and he reports his health perfect.

That hæmatogenous infections play a very important part in the etiology of cholecystitis with its resultant pathological disturbances must be considered. In the Southern States where a preponderance of malaria, dysentery and other bowl troubles besides yellow fever and autotoxæmias so prevail, an observing M. D. therein has a field for large opportunities to observe and work out the consequences of these disturbances. Hence the well equipped medical mind in the Southern States are prone to the closer observations of the liver and its addnexa.

Why do we find the terms bilious or torpid liver, bilious fever as almost household words in the low lands of the South? I can give no better explanation than the want of understanding of the damages done by infection and how the over-taxed liver to eliminate a system charged with infections and toxæmias manifest such symptoms that the laity has seen fit to dub the terms that are so superficial in their meaning. So frequently we see individuals manifesting lowered nervous energy disturbed functions of the alimentary canal, the appearance of the skin so suggestive, etc., all summed up by the term, malaise. Should a proper investigation of such a case be made, we will find most probably conditions existing, such as infections and auto-toxæmias as primary factors. Frequently where operations were made for gall stones, we learn none of these were found in the gall bladder, though we discover a cholecystitis and its complications present. The proper course of procedure to correct this cholecystitis and its adnexa disturbances which has been produced by the previous mentioned causes, has been with me after doing a cholecystotomy to proceed along the lines of post operative treatment as above given, for to do a cholecystotomy and simply drain the gall bladder without the essential post operative treatment is only to invite a failure.

The following case will serve to illustrate this point: Captain E. M., about 70 years old, had been eight years previously operated on for gall stones. For the past three years have been called frequently to see him for cricies of apparent gall stone colic. The examination of his skin wound and his statement showed no drainage tube and post operative treatment had been made when he was operated on. Palpation impressed me that there was a large and sensitive gall bladder. His attacks growing more severe, his general resistance was so lowered that he at last submitted to a second operative procedure. We found a very large gall bladder thickened and bent upon itself extending down over the region of the ascending colon. The attachments were broken up. This thickened gall bladder that was quite friable was brought up and emptied of quite a quantity of pernicious biliary fluid. Examination revealed no gall stones present, the cystic and common ducts probed out and found quite patulous. The condition warranted a cholecystectomy which was done, the patient made a rapid and satisfactory recovery. This patient had never given a history of jaundice.

An interesting case that came under my observation some years since was a cholecystectomy. After the drainage through the abdominal wound of infected bile ceased and the fistule had closed, soon resulted a disturbance similar to what he had experienced prior to his operation. He submitted to another operation wherein I was informed that the gall bladder was anastomosed to the transverse colon. Resultant conditions that deprived the smaller intestines of the needed functions from the common duct were most distressing. After this the patient came under my care.

Mr. P. was rapidly losing his vitality. Having informed me that there were no gall stones found in either of the former operations, my efforts to combat the lack of the secretions from the common bile duct were successfully met by substituting as near as possible the much needed lost secretions together with the consideration of his dietery. I could but attribute that the trouble in this patient was due to an obstructive bile duct, the trauma of the gall stones that had previously passed therethrough. Three years since this treatment was made, the patient quickly went on to a satisfactory recovery and resumed his business callings.

Typhoid fever that is often so insidious and so tenacious in its far-reaching sequelæ commands high consideration in our thoughts of the etiology and sequelæ of cholecystitis. Will only cite a few cases wherein typhoid infections play an important part.

In 1900 was called to Washington, D. C., to diagnose, if not able to remain there, and treat a case that caused much anxiety. Mrs. S., age about 38, after several recurrent attacks was found to be in a state of rather severe illness. The symptoms seemed to point to recurrent gastritis with its complications. Upon examination, my opinion was that this was a case of chronic cholecystitis with its various disturbances of gall stones, etc. The history gave an attack of typhoid fever when she was 16 years old. Operation of cholecystectomy was done. Gall stones were found, and post operative treatment made. This patient has made a very satisfactory recovery, having enjoyed perfect health since. One of these gall stones was submitted to the pathological department of Johns Hopkins University. The findings therefrom reported typhoid bacilli as the nucleus of this stone.

Another very interesting case that shows how important it is to consider the question of reflex abdominal pains, I will give. Six years ago a Mr. D., of Nevada, well advanced in years, a mining man, gave a history of nutritive disturbances referred and treated for several years for gastritis. Upon examination I diagnosed a case of cholecystitis with gall stones and their sequelæ. This patient was very skeptical as to gall stones, as he had never experienced a state of jaundice. His history gave an attack of typhoid fever many years previous. Operative procedure revealed many gall stones with thickened gall bladder. Within three weeks he returned to his home in Nevada. I have heard from him repeatedly to the effect that he has no further stomach trouble and his general vigor is fully restored.

Typhoid fever with its hæmatogenous infections I found to be a very important etilogical factor in the consideration of cholecystitis and its complications. Appendicitis, as a cause of cholecystitis, must not be disregarded. With appendicitis resultant hæmatogenous infections follow also the natural process of eliminating the blood disturbances. It has been claimed that as high as 80% of cholecystitis arises from appendicitis. After due deliberation it is well for us whose work it is to aid violated nature or prevent illness, to consider how hæmatogenous infections arise and how to prevent them. Pain in the epigastric region is as misleading to the doctor, as well as the patient, in reaching a diagnosis of gall stone colic. The following case of many will demonstrate this point:

Mrs. F. consulted me for what she told me were attacks of acute indigestion that had been distressing her for many years, so that she carried a bottle of medicine along with her wherever she went to relieve the cricises. My diagnosis differentiated down to a case of cholecystitis, etc. The patient tabooed this diagnosis. She had never had an attack of jaundice. A few days after I was called and found an impacted gall stone in the common bile duct with its full train of concomitant symptoms. Consultations confirmed my diagnosis. Cholecystectomy was done. Many gall stones removed. Post operative treatment, etc., carried out, result, complete cure.

Many such cases as this I could give from my personal experience to illustrate how essential the close differential observations for cholecystitis should be regarded. The advanced results attained by our research workers along the lines of infections and toxæmias have aroused the surgeon's consideration to their value. We have learned that the liver bears the greater tax in its effort to eliminate infections from the blood. Here is where our surgical procedures, if properly considered in our ante and post operative work has recently made, is making, and will make, most satisfactory returns in attaining better results for our patients.

The becoming consideration of the alimentary canal, as well as the hæmatogenous infections must as far as possible engage the surgeon's attention ere operating. First, in preparing our patient for an operation, we should give due regard to the alimentary canal. This is the source of the majority of auto-toxæmias; it is also advantageous to use hexamethylamin. (The best preparation of this I believe to be Hemitol of Fried & aByer.) The object of this drug as an antiseptic is evident, using this both ante and post operative. The administration of Neiser's mixed bacteria I deem worthy also of ante operative consideration.

Recently I was much strengthened in my views by a paper read before the Medical Society of London, by W. A. Lane, surgeon to Guys Hospital, on chronic intestinal stasis, wherein he lays great stress upon intestinal infections and how varied and far-reaching are the symptoms therefrom. He cites many interesting cases where cures were obtained by eliminating this source of poison. I can but commend in our surgical work the considerartion of both the ante and post operative treatment in dealing with cholecystitis.

